# Combined Effects of Diet and Physical Activity on Inflammatory Joint Disease: A Systematic Review and Meta-Analysis

**DOI:** 10.3390/healthcare11101427

**Published:** 2023-05-15

**Authors:** Petros C. Dinas, on behalf of the students of module 5104 (Introduction to Systematic Reviews), Rikke Helene Moe, Carina Boström, Rena I. Kosti, George D. Kitas, George S. Metsios

**Affiliations:** 1 Department of Nutrition and Dietetics, School of Physical Education, Sport Science and Dietetics, University of Thessaly, 42130 Trikala, Greece; renakosti@uth.gr (R.I.K.); g.metsios@uth.gr (G.S.M.); 2 FAME Laboratory, School of Physical Education, Sport Science and Dietetics, University of Thessaly, 42131 Trikala, Greece; 3 National Resource Centre for Rehabilitation in Rheumatology, Department of Rheumatology, Diakonhjemmet Hospital, 0370 Oslo, Norway; rikmoe@gmail.com; 4 Department of Neurobiology, Care Sciences and Society, Division of Physiotherapy, Karolinska Institutet, 14183 Huddinge, Sweden; carina.bostrom@ki.se; 5 Dudley Group of Hospitals NHS Foundation Trust, Department of Rheumatology, Russells Hall Hospital, Dudley DY1 2HQ, UK; george.kitas@nhs.net; 6 School of Sport, Exercise and Rehabilitation Sciences, University of Birmingham, Birmingham B15 2SQ, UK

**Keywords:** rheumatoid arthritis, spondyloarthropathies, HAQ, DAS28

## Abstract

Research evidence suggests that, individually, diet and physical activity are effective interventions for reducing levels of inflammation in inflammatory joint diseases (IJD), however little is known about their combined use. This systematic review and meta-analysis aimed to examine the effects and/or associations of combined diet and physical activity interventions in IJD, specifically rheumatoid arthritis (RA) and the spondyloarthropathies (SpA) (PROSPERO registration number: CRD42022370993). Ten out of 11 eligible studies examined RA patients. We found that a combination of diet/nutrition and physical activity/exercise improved Health Assessment Questionnaire score (standardized mean difference = −1.36, confidence interval (CI) = (−2.43)−(−0.30), I^2^ = 90%, Z = 2.5, *p* = 0.01), while surprisingly they increased erythrocyte sedimentation rate (mean difference = 0.20, CI = 0.09–0.31, I^2^ = 0%, Z = 3.45, *p* < 0.01). No effects were found on C-reactive protein or weight (*p* > 0.05) of RA patients. We did not find studies in other IJDs that provided sufficient data for a meta-analysis. The narrative data synthesis provided limited evidence to address our research question. No firm conclusions can be made as to whether the combination of diet/nutrition and physical activity/exercise affects inflammatory load in IJDs. The results of this study can only be used as a means of highlighting the low-quality evidence in this field of investigation and the need for further and better-quality research.

## 1. Introduction

Inflammatory joint diseases (IJD) are a cluster of non-communicable auto-immune disorders characterized by increased expression of inflammatory cytokines and an acute phase response (such as high C-reactive protein—CRP or erythrocyte sedimentation rate —ESR). If not controlled, this leads to specific and persistent symptomatology, including joint pain, fatigue, and eventual joint destruction and functional disability, thus significantly affecting the quality of life of people living with IJD [1,2]. Amongst IJDs, the most prevalent and extensively studied are rheumatoid arthritis (RA) and the spondyloarthropathies, such as axial spondyloarthritis (AxSpa) (including ankylosing spondylitis (AS)) and psoriatic arthritis (PsA).

Several pharmaceutical interventions are available for alleviating the symptoms and improving outcomes in IJDs, all aiming to achieve remission via targeting different inflammatory pathways. Increased usage of biologic (e.g., tumor necrosis factor alpha (TNFa) inhibitors) or targeted synthetic disease-modifying antirheumatic agents (DMARDs) has improved outcomes but tripled the costs of healthcare in IJDs [3]. It has also uncovered the problem of multi-morbidity associated with IJDs. For example, in most of these conditions, there is a high prevalence of traditional cardiovascular disease (CVD) risk factors, such as hypertension [4,5], dyslipidemia [6], obesity [7], vascular dysfunction [8,9], and type II diabetes [10] contributing to a higher prevalence and worse CVD outcomes [11]. Such associations strongly suggest the necessity of a multidisciplinary approach in the management of IJD both at individual and healthcare level, as evidenced in recent European Alliance of Associations for Rheumatology (EULAR) recommendations [11].

Research evidence suggests that diet and physical activity, used individually, are effective interventions for reducing levels of inflammation. Diets such as the Mediterranean diet, contain anti-inflammatory nutrients (e.g., polyphenols and anti-oxidants), which collective epidemiological data associate with reduced inflammation [12]. On the other hand, physical activity can reduce inflammation acutely via promoting an anti-inflammatory phenotype, while in the long run the reduction in inflammation is mediated by the exercise-induced reductions in adipose tissue mass [13]. Therefore, the combination of these interventions may be important as an adjunct treatment for alleviating the inflammatory load in IJD. The aim of this systematic review and meta-analysis, was to examine the effects (from interventional studies) and/or associations (from epidemiological studies) of combined diet and physical activity interventions on IJD.

## 2. Materials and Methods

We conducted a systematic review and meta-analysis following the Preferred Reporting Items for Systematic Reviews and Meta-analyses (PRISMA) guidelines [14] and the Cochrane handbook [15]. Our study was registered with the International Prospective Register of Systematic Reviews (PROSPERO) database; registration number: CRD42022370993 [16].

### 2.1. Searching and Selection Processes

Three databases, PubMed, EMBASE, and SportDiscus, were searched up until October 2022. No restrictions were applied regarding date and language of publication, or study design. Search algorithms are shown in the Supplementary Materials. The students who are listed as co-authors herein, formed the searching algorithm, while the searching procedure was performed by PCD and checked by GSM and GDK. The students then formed eight teams and performed the selection of the eligible studies. Each team of students screened approximately 60 retrieved papers against the eligibility criteria. PCD independently screened all retrieved papers. Any disagreement between the teams of students and PCD were resolved through a referee investigator (GSM). Finally, PCD and RHM performed the reference lists screening of the eligible publications, to identify additional eligible publications that did not appear in the initial searching.

### 2.2. Study Inclusion and Exclusion Criteria

Studies that involved human participants were included in the present systematic review. Eligible studies included:

1. Randomized controlled trials (RCT), quasi RCT, controlled trials (CT), and single arm design studies that integrated: (a) combined interventions of any diet/nutrition and any physical activity/exercise, AND (b) were performed on RA patients and/or SpA patients AND (c) participants of any ethnicity, age, and gender, AND (d) participants under any pharmacological treatment. As a control situation, we accepted RCT, quasi RCT, and CT that used a control group (i.e., usual care) as well as single arm design studies that used baseline measurements as a control situation that were compared with post intervention measurements.
2. Epidemiological studies that integrated: (a) combined measurements of diet/nutrition and physical activity/exercise, AND (b) were performed on RA patients and/or SpA patients, as well as in the general population (adults) to explore associations with the above-mentioned diseases, AND (c) participants of any ethnicity, age, and gender, AND (d) participants under any pharmacological treatment.
3. Animal studies, reviews, study protocols, editorials, conference proceedings, magazines, and grey literature publications were excluded.

### 2.3. Study Quality Assessment

For the eligible RCT, we used the updated Risk of Bias 2 (RoB2) Cochrane library tool [17] and for the quasi RCT, CT, as well as the epidemiological eligible studies we used the Research Triangle Institute Item Bank (RTI-IB) tool [18], to assess their risk of bias. The team of students and PCD as well as CB, independently performed the risk of bias assessment. Each team assessed the risk of bias in one or two eligible papers, while PCD and CB assessed the risk of bias in all eligible papers. GSM acted as a referee in case of disagreement.

### 2.4. Data Extraction Strategy

We extracted data from the eligible studies as follows: (a) first author surname and year of publication, (b) methodological design (i.e., RCT, quasi RCT, CT or epidemiological), (c) participants’ anthropometric characteristics (i.e., age, gender, body mass index (BMI)), (d) participants’ disease characteristics (i.e., disease activity, duration, etc.), (e) physical activity/exercise interventions/measurements, (f) diet/nutrition interventions/measurements, and (g) main outcome. The extracted data can be found in Supplementary Materials, Table S1. The data extraction method included an initial calibration within the classroom on which data were extracted and details on which data extraction form should be used. Accordingly, each team of students extracted data from one or two eligible papers, while PCD, ΡΙΚ, and CB extracted data from all eligible papers, independently. GSM and GDK acted as referees in case of disagreements.

### 2.5. Data Synthesis and Presentation

For eligible studies that did not provide numerical data to be used for a meta-analysis, a summarized narrative data synthesis was adopted. For studies suitable for meta-analysis, a random-effect model was used to account for heterogeneity due to differences in study populations, disease characteristics, diet/nutrition and physical activity/exercise interventions/measurements, and study duration. All meta-analyses were conducted using RevMan 5.4.1, 2020 software (The Cochrane Collaboration, Oxford, UK) [19]. We used an inverse variance, continuous method to calculate mean differences (MD) either between a combined intervention of diet/nutrition and physical activity/exercise and a control group, or between baseline and post intervention values. In cases where calculation units were different for the variable involved in a meta-analysis, a standardized mean difference (SMD) approach was used [15]. We identified four main variables suitable for meta-analysis: weight (kg), Health Assessment Questionnaire (HAQ) score, erythrocyte sedimentation rate (ESR; mm/1st h), and CRP (mg/L). The HAQ represents patients-oriented outcome assessment, which is based on five dimensions: disability, pain, medication effects, costs of care, and mortality [20].

For one eligible study [21], we converted the standard error into standard deviation (SD) using the equation: $\mathrm{SD}=standard\;error\times \sqrt{n}$ [15]. For two eligible studies [22,23], we calculated the means and SD from medians and the 1st–3rd quartiles, according to: *mean* = (*q1* + *m* + *q3*)/3; SD = (*q3* − *q1*)/1.35 [15,24], while for two eligible studies [21,25], we calculated SD from *p* values of the performed *t*-tests, using the following equation in Excel: SD = *TINV* [(*p value*; (*N1* + *N2*) − 2] [15]. As non-parametric and parametric data cannot be mixed in a meta-analysis [15], we converted the means and SD of parametric data of one eligible study [25] into non-parametric data, using well-established equations [26]: *mean* $=\frac{1}{2}ln(\frac{S}{X}$ + 1); SD = $\sqrt{\ln\left(\frac{S}{X}+1\right)}$ [26].

### 2.6. Evidence of Effectiveness

We evaluated the quality of evidence of each meta-analysis via the Grading of Recommendations Assessment, Development and Evaluation (GRADE) analysis [15,27].

## 3. Results

The reporting information of the current systematic review and meta-analysis can be found in a relevant PRISMA checklist (Supplementary Materials, Table S3).

### 3.1. Results of Searching and Selection Processes

The searching procedure retrieved 854 publications, of which 257 were duplicated, leaving 597 publications for eligibility screening. From those 597 publications, 418 were reviews, editorials, case reports, consensus papers, and conference proceedings and were excluded during the first round of the selection process. From the remaining 179 publications, 166 were excluded as non-eligible, one was excluded because it was not possible to locate the full text given that the journal of publication was discontinued and no archive existed, and one publication was excluded due to containing secondary data from an already eligible RCT. Finally, 11 publications were eligible and included in the current systematic review. The screening of the reference lists of these 16 eligible papers, revealed no more eligible publications. A relevant PRISMA flow diagram can be found in Supplementary Figure S1.

### 3.2. Characteristics of Included Studies

Three of the included studies in the current systematic review were RCT [23,25,28], three were CT [21,29,30], and the remaining studies were epidemiological. Ten studies referred to RA patients [21,22,23,25,28,30,31,32,33,34], while two studies [29,31] referred to SpA. One study was an epidemiological design study that used a sample of the general population to identify associations of the combination of diet/nutrition and physical activity with self-reported RA [33]. Overall, the included studies in the current systematic review involved 495 RA patients, 22 SpA patients, and 11,768 participants from the general population.

### 3.3. Study Quality Assessment Outcomes

For the randomization process, all the eligible RCTs displayed some concerns for risk of bias; for intervention assignment one RCT [28] demonstrated some concerns and the remaining two [23,25] low risk of bias. For intervention adherence, one RCT [25] displayed some concerns and the other two [23,28] low risk of bias. For missing data and outcome risk of bias all RCTs demonstrated low risk, while for reported results one RCT [25] displayed some concerns and the other two [23,28] demonstrated low risk of bias.

**Figure 1:**
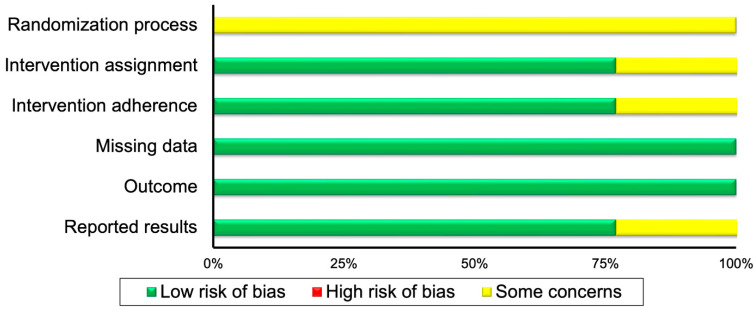
Summary of risk of bias for randomized controlled trials.

**Figure 2:**
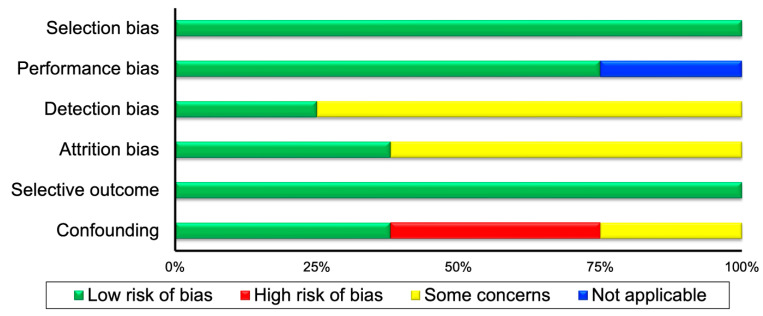
Summary of risk of bias for controlled trials and epidemiological studies.

Regarding the CT and epidemiological included studies, in the selection bias all studies demonstrated low risk. For performance bias, two studies [31,33] were not applicable and six studies [21,22,29,30,32,34] displayed low risk of bias. For detection bias, two studies [30,33] displayed low, and the remaining six, some risk of bias concerns. In the attrition bias, three studies [21,29,30] demonstrated low, and the remaining five, some risk of bias concerns. In the selective outcome, all studies demonstrated low risk of bias. Finally, in the confounding risk, three studies [22,29,31] displayed high risk, three [21,30,32] demonstrated low risk, and two studies some risk of bias concerns. The risk of bias outcomes can be found in Table 1, while a summary of the risk of bias can be found in Figure 1 for RCT and in Figure 2, for the CT and epidemiological studies.

### 3.4. Narrative Data Synthesis Results

To adequately synthesize the evidence, we created a table and a heat-map (Table 2). Most of the eligible studies included in the current systematic review examined RA patients; only two studies examined SpA patients (one study PsA and one study AS) [29,31], respectively.

#### 3.4.1. Narrative Data Synthesis Results for Controlled Trials

One CT revealed that a combination of a 12-week resistance exercise program and a reduction of 30% of energy intake, as well as 62 gr of high-quality protein intake per day, vitamins, and mineral supplements, reduced body fat mass and fat free mass in RA patients [21]. Likewise, another CT showed that a combination of a protein drink and an acute exercise program, stimulated muscle protein synthesis and transcriptional regulation in RA patients to a similar degree as in healthy individuals [30]. Finally, a CT showed an improvement in quality of life and reduction in total fat mass (kg) when patients with PsA followed a 12-month physical activity (>150 min/week) and a weight loss treatment intervention with very low energy diet [29].

#### 3.4.2. Narrative Data Synthesis Results for Epidemiological Studies

One study revealed no association of physical activity and energy intake with HAQ, DAS28, body fat, BMI, interleukin-1β, interleukin-6, TNFa, ESR, and CRP, in RA patients [34], while for RA and AS patients, one study showed no associations of physical activity levels and daily calorie as well as protein intake with sarcopenia [31]. For RA patients, a study demonstrated no association of healthy eating index total scores with self-reported RA, adjusted for physical activity [33]. Finally, a study revealed that RA patients who consumed saturated fatty acids and had low levels of total physical activity displayed significantly lower levels of high-density lipoprotein, apolipoprotein A1, atheroprotective anti-phosphorylcholine antibodies, and significantly higher levels of insulin [32].

### 3.5. Meta-Analyses Outcomes

**Figure 3:**
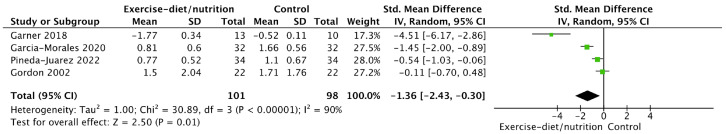
Forest plot of the effect of combined intervention of diet/nutrition and physical activity/exercise on HAQ score in RA patients [22,23,25,28].

For HAQ score, a meta-analysis showed that RA patients that undertook an intervention of diet/nutrition and physical activity/exercise displayed lower HAQ score than control participants (SMD = −1.36, confidence interval (CI) = (−2.43)−(−0.30), I^2^ = 90%, Z = 2.5, *p* = 0.01, Figure 3).

**Figure 4:**
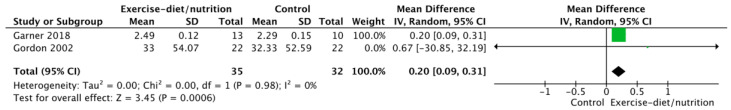
Forest plot of the effect of combined intervention of diet/nutrition and physical activity/exercise on ESR (mm/1st h) in RA patients [22,25].

We found that ESR (mm/1st h) is increased as a result of a combined intervention of diet/nutrition and physical activity/exercise (MD = 0.20, CI = 0.09–0.31, I^2^ = 0%, Z = 3.45, *p* < 0.01, Figure 4), in RA patients.

Two studies that were performed in RA patients offered data to conduct a meta-analysis of MD in weight (kg), between an intervention of a combination of diet/nutrition and physical activity/exercise and no intervention. The outcome showed no differences (*p* > 0.05, Supplementary Figure S2). Finally, we found that CRP (mg/L) is not affected by a combined intervention of diet/nutrition and physical activity/exercise, in RA patients (*p* > 0.05, Supplementary Figure S3).

### 3.6. GRADE Analysis Outcomes

The GRADE analysis revealed low quality evidence for the meta-analyses conducted (Table 3) due to inconsistency of results and due to imprecision. The detailed analysis can be found in the Supplementary Materials, Table S2. Regarding the meta-analysis on HAQ score, the studies included displayed high heterogeneity (I^2^ = 90%). Even though we used a random effect model meta-analysis, which may ignore heterogeneity [15], heterogeneity (i.e., any kind of variability among the included studies) is an important element to consider especially when a small number of studies is included [15], and therefore, we downgraded the quality of evidence for inconsistency [15,27]. Concerning the meta-analysis on ESR, the studies included showed an overall sample size of 67 patients, which does not meet the optimal information size requirements of GRADE, and therefore, we have downgraded the quality of evidence for imprecision [15,27].

## 4. Discussion

### 4.1. Summary of Main Findings

The aim of the current systematic review and meta-analysis was to examine the effects (from interventional studies) and/or associations (from epidemiological studies) of combined diet and physical activity interventions on IJDs. Overall, we cannot make robust conclusions based on the available evidence in IJDs. Our meta-analyses revealed that a combination of diet/nutrition and physical activity/exercise was associated with improved HAQ score but also with increased ESR, in RA patients. No effect of a combined intervention of diet/nutrition and physical activity/exercise on CRP and weight (kg) of RA patients was found. The narrative data synthesis for controlled trials showed improvement in quality of life for PsA patients [29] and reduced body fat mass and fat free mass for RA patients [21], after a 12-month and a 12-week intervention, respectively. Furthermore, RA patients stimulated muscle protein synthesis and transcriptional regulation to a similar degree as in healthy individuals, after a combination of a protein drink and an acute exercise program [30]. The narrative data synthesis for three epidemiological studies [31,33,34] revealed no association of a combination of diet/nutrition and physical activity/exercise with several RA indices, while three epidemiological studies [21,30,32] showed that a combination of diet/nutrition and physical activity/exercise may improve RA indices.

### 4.2. Completeness and Applicability of Evidence

We found very limited evidence to examine our research question in SpA. Therefore, our systematic review mainly provides findings related to RA. For RA patients, there was a large heterogeneity of the diet/nutrition and physical activity/exercise interventions and associations examined as well as in the investigated outcomes. One of our meta-analyses showed that a combined intervention of diet/nutrition and physical activity/exercise reduces HAQ score, thus indicating improvement in physical functioning in people living with RA. This outcome, however, should be treated with caution, given the low quality of evidence that our GRADE analysis revealed. The interventions involved in this meta-analysis included a combination of exercise (aerobic and resistance) and Mediterranean diet as well as advice to perform adequate physical activity and follow a healthy diet. This outcome is in line with the overall notion that diet/nutrition and physical activity/exercise can improve RA patients’ quality of movement, as this is expressed in guidelines and position statements [35,36]. Specifically, consumption of fish oil/omega-3, probiotics, vitamin D, B16 and E, as well as antioxidants was shown to improve CRP, pain, functional ability, and disease activity [35], while vegetarian and/or vegan diets may be more beneficial for reducing body weight than conventional calorie-restricted diets, in RA patients [37,38]. Physical activity recommendations include 3000 steps/day of moderate intensity, while for sedentary, low, and somewhat physically active RA patients, the target should be 10,000 steps/day [39].

Increasing physical activity in RA patients may also be beneficial in fall risk, given that RA gait disturbances may occur [40]. Indeed, balance disturbances in RA patients are positively associated with a risk of falls of 33% [41,42]. Furthermore, lack of physical activity in RA patients lowers their functional ability by approximately 60% in females and 40% in males [40]. Similarly, RA patients may experience sarcopenia (approximately 3–24%), which is associated with falls and poor functional ability [43]. Nutritional treatments are suggested for sarcopenia [44], indicating the role of diet in reducing functional ability including gait disturbances in patients with RA.

Furthermore, one of the meta-analyses found low quality evidence that ESR is increased due to the combination of diet/nutrition and physical activity/exercise, in RA patients. The interventions involved in this meta-analysis included a combination of exercise or advice to perform adequate physical activity and counselling regarding a healthy diet. The finding indicates a potentially harmful effect of the combination of these interventions as per the observed increase in the inflammatory load in RA. To the best of our knowledge our meta-analysis is the first to examine the combined effect of diet/nutrition and physical activity/exercise on ESR of RA patients, therefore, there is no previous meta-analysis to compare our finding to. Furthermore, the two eligible studies included in our meta-analysis found no statistically significant differences in ESR between experimental and control groups [25], or baseline and post intervention [22] measurements, thus they provide no explanation about the ESR trend due to their interventions. The two eligible studies included in our meta-analysis also found no statistically significant differences in CRP, therefore, the outcome for ESR may be an odd random result. Regarding exercise, a previous meta-analysis showed that it decreased ESR [45], while several diet schemes are able to reduce ESR in RA patients [46]. Therefore, it is likely that our finding, i.e., the combined effect of diet and physical activity, which is based on a small number of studies, can be random. Given the biological plausibility of both physical activity and diet to reduce inflammatory load, more and better-quality studies are currently required to determine their combined effects on inflammation and other important clinical outcomes in IJDs. It is well-documented though that adequate physical activity/exercise can improve systemic manifestations of RA [39,45,47].

The narrative data synthesis revealed an association between RA indices and the combinations of adequate physical activity or exercise participation and increased protein intake [21,30]. While some investigators did not confirm an association of protein consumption with RA [48,49], a recent systematic review also showed that products containing high quality proteins (i.e., meat) did not associate with RA [50]. It is therefore likely that the association found in our systematic review may be attributed solely to the favorable effect of physical activity. Finally, the narrative data synthesis also revealed studies that failed to connect the combination of diet/nutrition and physical activity/exercise with RA.

### 4.3. Strengths and Potential Biases in the Review Process

Our systematic review includes some strengths. We used suitable algorithms with standardized indexing terms in the search procedure, which may have retrieved publications that use alternative keywords to describe the same concept [15]. We also used robust searching and screening procedures, risk of bias assessment, and data extraction; we did not exclude studies based on language and time of publication. Finally, we evaluated the quality of our meta-analyses through GRADE analysis, which determined the quality of our findings.

Our systematic review also includes limitations. The sample size of the meta-analyses performed was small, in order to adequately answer our research question (i.e., low quality of evidence based on our GRADE analysis). Furthermore, there was a large heterogeneity regarding the population characteristics, disease activity, type of measurements, diet/nutrition, and physical activity/exercise schemes, which may have compromised the interpretation of our findings. However, our systematic review revealed the gaps in the current knowledge and may alert the scientific community to undertake further research on the topic, given the biological plausibility that both these interventions have in reducing inflammatory load. Finally, it was not possible to take into consideration the drugs received by the participants as part of their medical treatment, which may have affected the outcomes of the interventions in the eligible studies, and consequently our data synthesis outcomes.

### 4.4. Statement on Significant Deviations in Methods from the Published Protocol

We report no significant deviations from the published protocol [16].

## 5. Conclusions and Future Directions

We conclude that more targeted research of good methodological quality is necessary in IJDs. Most of the available studies were regarding RA, albeit with a high heterogeneity, which is not surprising given that this is the most well studied IJD. Given the results of our study, no firm conclusions can be made as to whether the combination of diet/nutrition and physical activity/exercise affects inflammatory load in IJDs. The results of this study can only be used as a means of highlighting the low-quality evidence in this field of investigation. We believe that this lack of evidence for the combined effects of diet/nutrition and physical activity/exercise on IJDs, is due to the complexity of potential interventions of diet/nutrition and physical activity, given their wide spectrum. This makes it difficult to draw meaningful conclusions. Another reason could be the complexity of designing such interventions, due to the national and regional population characteristics, healthcare systems, and rapidly changing characteristics of society.

Given the amount of available evidence in the current systematic review, it is obvious that the issue is not well-documented by previous research and therefore, we suggest the following: (a) new RCTs are needed to examine the effects of combined diet and physical activity interventions on IJDs, (b) new epidemiological studies are needed to identify the potential diet/nutrition and physical activity/exercise factors that play roles in the risk of IJDs, as well as the potential adverse effects, and (c) a personalized medicine data collection and analysis approach is needed, to provide information for stratification and individualization towards the treatments and prevention of IJDs. The combination of diet/nutrition and physical activity/exercise is included in the behavioral risk factors for IJDs, which however, may imply a personalized approach to be effective. It could therefore, necessitate that these behavioral data should be included in a wider personalized data collection approach—e.g., genomics, epigenomics, proteomics, foodomics, metabolomics, etc.—connected with IJDs. This approach could complement treatments for IJDs and public health prevention strategies.

## Figures and Tables

**Table 1:** Risk of bias assessment outcomes. Key: +: low (green color), ?: some concerns (yellow color), −: high (red color), N: not applicable (blue color).

Randomized Controlled Trials						
First author	Randomization process	Intervention assignment	Intervention adherence	Missing data	Outcome	Reported results
Garcia-Morales 2020 [28]	?	?	+	+	+	+
Garner 2018 [25]	?	+	?	+	+	?
Pineda-Juarez 2022 [23]	?	+	+	+	+	+
**Controlled trials and epidemiological studies**						
First author	Selection	Performance	Detection	Attrition	Selective Outcome	Confounding
Barone 2018 [31]	+	N	?	?	+	−
Bilberg 2022 [29]	+	+	?	+	+	−
Elkan 2011 [32]	+	+	?	?	+	+
Engelhart 1996 [21]	+	+	?	+	+	+
Gordon 2002 [22]	+	+	?	?	+	−
Matsunaga 2021 [33]	+	N	+	?	+	?
Mikkelsen 2015 [30]	+	+	+	+	+	+
Stavropoulos-Kalinoglou 2010 [34]	+	+	?	?	+	?

**Table 2:** Narrative data synthesis outcomes and heat-map for the eligible studies that are not included in a meta-analysis, or some of their outcomes could not be included in a meta-analysis; green color represents a positive effect or association; orange color represents no association: RA = rheumatoid arthritis; PsA = psoriatic arthritis; AS = ankylosing spondylitis; HAQ = health assessment questionnaire; DAS28 = disease activity score 28; BMI = body mass index; ESR = erythrocyte sedimentation rate; CRP = C reactive protein; HDL = high density lipoprotein.

Study	Type of Patients	Type of Diet/Nutrition and Exercise/Physical Activity	Outcome
**Controlled Trials**			
Engelhart, 1996 [21]	RA	12-week resistance exercise and walking. Reduction of 30% of energy intake, 62 gof high-quality protein intake per day, vitamins, and mineral supplements	Reduction of body fat mass and fat free mass
Mikkelsen, 2015 [30]	RA	A protein drink consisting of 0.5 g intact whey protein isolate (Lacprodan-9224, Arla Foods Ingredients, Viby, Denmark)/kg lean body mass (12.5% enriched with ring-13C6-phenylalanine) dissolved in 190 mL water. Acute exercise of unilateral leg extension, 8 × 10 repetitions at 70% of 1 repetition maximum	Muscle protein synthesis and transcriptional regulation can be stimulated with both protein intake and physical exercise in patients with RA to a similar degree as in healthy individuals
Bilberg, 2022 [29]	PsA	Recommendation for physical activity, ≥150 min/week and weight loss treatment	Patients improved quality of life (SF-36 score), total fat mass (kg), and reduced total lean mass from baseline to 6-month (*p* < 0.01) and 12-month (*p* < 0.01).
**Epidemiological studies**			
Elkan, 2011 [32]	RA	Questionnaires assessed dietary habits and physical activity levels	RA patients who consumed saturated fatty acids and had low level of total physical activity displayed significantly lower levels of HDL, apolipoprotein A1, the atheroprotective anti-phosphorylcholine antibodies, and significantly higher levels of insulin
Barone, 2018 [31]	RA	Measurements of physical activity and daily calorie and protein intake	No association of physical activity and daily calorie and protein intake with sarcopenia in patients
Matsunaga, 2021 [33]	RA	Questionnaires assessed dietary habits and physical activity levels	No association of healthy eating index total scores with self-reported RA, adjusted for physical activity
Stavropoulos-Kalinoglou, 2010 [34]	RA	Questionnaires assessed dietary habits and physical activity levels	Physical activity and energy intake were not associated with HAQ, DAS28, body fat, BMI, interleukin-1β, interleukin-6, tumor necrosis factor alpha, ESR, and CRP
Barone, 2018 [31]	PsA	Measurements of physical activity and daily calorie and protein intake	No association of physical activity and daily calorie and protein intake with sarcopenia in patients
Barone, 2018 [31]	AS	Measurements of physical activity and daily calorie and protein intake	No association of physical activity and daily calorie and protein intake with sarcopenia in patients

**Table 3:** GRADE analysis outcomes.

Outcomes	No of Participants (Studies/Entries)	Quality of the Evidence (GRADE)	Relative Effect (95% CI)
Diet/nutrition and physical activity/exercise HAQ score vs. control	199 (4 studies/entries)	Low ⊕⊕◯◯ due to inconsistency of results	Standardized mean difference = −1.36 (−2.43)–(−0.30)
Diet/nutrition and physical activity/exercise ESR vs. control	67 (2 studies/entries)	Low ⊕⊕◯◯ due to imprecision	Mean difference = 0.2 (0.09–0.31)

## Data Availability

Extracted data used in the meta-analyses are available upon reasonable request.

## Supplementary Materials

The following supporting information can be downloaded at: https://www.mdpi.com/article/10.3390/healthcare11101427/s1, Table S1: Characteristics of the eligible studies (extracted data); Table S2: Detailed GRADE analysis for the meta-analyses performed; Table S3: PRISMA checklist.; Figure S1: PRISMA flow diagram; Figure S2: Forest plot of the effect of combined intervention of diet/nutrition and physical activity/exercise on weight (kg), in RA patients.; Figure S3: Forest plot of the effect of combined intervention of diet/nutrition and physical activity/exercise on CRP (mg/L), in RA patients.

## Appendix A

List of students who meet the criteria for co-authors, in alphabetical order.

	**Full Name**	**Email**
1	Adriana Machaira	amachaira@uth.gr
2	Aggelos Mpaltos	abaltos@uth.gr
3	Anastasia Grigoriadou	anasgrigoriadou@uth.gr
4	Anastasia Prokopiou	anprokopiou@uth.gr
5	Anastasia-Foteini Mpanti	anbanti@uth.gr
6	Anna Daskalaki	anndaskalaki@uth.gr
7	Anna-Maria Stamathioudaki	astamath@uth.gr
8	Anthi Karambelia	akarampelia@uth.gr
9	Anthi Kousi	antkousi@uth.gr
10	Argyri Nerantzoglou	anerantzoglou@uth.gr
11	Antonia Daskalaki	andaskalaki@uth.gr
12	Antonia-Kiriaki Kampioti	akampioti@uth.gr
13	Athanasia-Vakchi Miliou	atmiliou@uth.gr
14	Athina-Efraimia Papoutsaki	apapoutsaki@uth.gr
15	Christina Mikelopoulou	cmikelopoulou@uth.gr
16	Christina Strotou	cstrotou@uth.gr
17	Chrysoula Psifogianni	cpsifogianni@uth.gr
18	Despoina Pantazelou	dpantazelou@uth.gr
19	Dimitra Falkou	dfalkou@uth.gr
20	Dimitra Theodorou	dimtheodorou@uth.gr
21	Dimitra Zapounidou	dzapounidou@uth.gr
22	Dimitra-Evdokia Kelepouri	dikelepouri@uth.gr
23	Dimitrios Marinakis	dmarinakis@uth.gr
24	Dimitrios-Ioannis Kasartzian	kasartziandimitris@gmail.com
25	Dimitrios-Paisios Koungoulas	dkougkoulas@uth.gr
26	Eleni-Anna Tsima	anntzima@uth.gr
27	Eleftheria-Paraskevi Sinani	elsinani@uth.gr
28	Eleftheria Thomaidi	elthomaidi@uth.gr
29	Elem Agko	eagko@uth.gr
30	Eleni Mpalasopoulou	ebalasopoulou@uth.gr
31	Eleni Tsironi	eletsironi@uth.gr
32	Eleni-Anna Tsavou	etsavou@uth.gr
33	Evaggelia-Tsampika Athanasa	eathanasa@uth.gr
34	Evaggelia-Christina Papadopoulou	evpapadopoul@uth.gr
35	Georgia Georgiou	georgiageo@uth.gr
36	Georgia Lavasa	glavasa@uth.gr
37	Georgia Loupsati	gloupsati@uth.gr
38	Georgia Tsakalou	gtsakalou@uth.gr
39	Georgios Charmantzis	gcharmantzis@uth.gr
40	Galatiani-Maria Fragkou	gafragkou@uth.gr
41	Ilia-Anna Verdou	iverdou@uth.gr
42	Ioanna Chatzigianni	ichatzigianni@uth.gr
43	Ioanna Gialou	igialou@uth.gr
44	Ioanna Goula	ioangoula@uth.gr
45	Katherina Karampet	kkarampet@uth.gr
46	Kyriaki Gyltidou	kgyltidou@uth.gr
47	Klaountio Ntemai	kntemai@uth.gr
48	Konstantina Kaziani	kkaziani@uth.gr
49	Maria Damala	mdamala@uth.gr
50	Maria Kosmatou	mkosmatou@uth.gr
51	Maria Mavreli	mmavreli@uth.gr
52	Maria Moschopoulou	mmoschopoulou@uth.gr
53	Maria Ntalouka	mantalouka@uth.gr
54	Maria-Niki Daniil	mardaniil@uth.gr
55	Marianna Grigoriadi	mardaniil@uth.gr
56	Marianna Nikoletopoulou	mnikolet@uth.gr
57	Marios-Ioannis Kyrilis	mkyrilis@uth.gr
58	Melek Kalentzi	mkalentzi@uth.gr
59	Nikoleta Adamidi	niadamidi@uth.gr
60	Nina Michailidou	nmichailidou@uth.gr
61	Ntorina Chotza	ntchotza@uth.gr
62	Olympios Stergiou	olstergiou@uth.gr
63	Panagiota Chatzoudi	pchatzoudi@uth.gr
64	Panagiota Karamvasi	pkaramvasi@uth.gr
65	Panagiota Nasopoulou	pnasopoulou@uth.gr
66	Panagiotis Efthimiadis	paefthymiadis@uth.gr
67	Paraskevi Apostolou	paapostolou@uth.gr
68	Philipos Papathemistokleous	fpapathem@uth.gr
69	Rentona Nourtsia	rnourtsia@uth.gr
70	Sofia Chatzivasileiou	sofichat@uth.gr
71	Sofia Karava	skarava@uth.gr
72	Sofia Panidou	sopanidou@uth.gr
73	Spyridon Stathopoulos	spstathopoulos@uth.gr
74	Stamatia Tzima	stamatiatsima@uth.gr
75	Stamatina Matiatou	smatiatou@uth.gr
76	Stamatis Vragkas	svragkas@uth.gr
77	Styliani Ferentinou	sferentinou@uth.gr
78	Theodora Fragista	tfragkista@uth.gr
79	Theodora-Iouliana Cojocariu	tcojocariu@uth.gr
80	Theodora Parpa	tparpa@uth.gr
81	Thiresia Chondrou	tchondrou@uth.gr
82	Thomais Karanika	thkaranika@uth.gr
83	Vaia Ntinti	vantinti@uth.gr
84	Vasiliki Doulaki	vdoulaki@uth.gr
85	Vasiliki Vythoulka	vvythoulka@uth.gr
86	Violeta Dionisidou-Konstantinidou	vdionysid@uth.gr
87	Vivjana Moulliri	vmoulliri@uth.gr
