# Supplementary material for: Combined Effects of Diet and Physical Activity on Inflammatory Joint Disease: A Systematic Review and Meta-Analysis
Source: Healthcare (Basel). 2023 May 15;11(10):1427. doi: 10.3390/healthcare11101427 (PMC10218217; doi:10.3390/healthcare11101427)
Supplement: Supplementary file 1 [file healthcare-11-01427-s001.zip › healthcare-2342809-supplementary.pdf]

SUPPLEMENTARY MATERIALS

Combined Effects of Diet and Physical Activity on Inflammatory Joint Disease: A Systematic Review and Meta-Analysis

Petros C. Dinas [1,2,*], on behalf of students of module 5104 (Introduction to Systematic Reviews) [1,†], Rikke Helene Moe [3], Carina Boström [4], Rena I. Kosti [1], George D. Kitas [5,6] and George S. Metsios [1]

[1] Department of Nutrition and Dietetics, School of Physical Education, Sport Science and Dietetics, University of Thessaly, 42130 Trikala, Greece; renakosti@uth.gr (R.I.K.); g.metsios@uth.gr (G.S.M.)
[2] FAME Laboratory, School of Physical Education, Sport Science and Dietetics, University of Thessaly, 42131 Trikala, Greece
[3] National Resource Centre for Rehabilitation in Rheumatology, Department of Rheumatology, Diakonhjemmet Hospital, 0370 Oslo, Norway; rikmoe@gmail.com
[4] Department of Neurobiology, Care Sciences and Society, Division of Physiotherapy, Karolinska Institutet, 14183 Huddinge, Sweden; carina.bostrom@ki.se
[5] Dudley Group of Hospitals NHS Foundation Trust, Department of Rheumatology, Russells Hall Hospital, Dudley DY1 2HQ, UK; george.kitas@nhs.net
[6] School of Sport, Exercise and Rehabilitation Sciences, University of Birmingham, Birmingham B15 2SQ, UK
* Correspondence: petros.cd@gmail.com
† A full list of the co-authors is provided in Appendix A.

KEY WORD ALGORITHMS

PubMed: ((((physical activity intervention*[Title/Abstract]) OR (physical activit*[Title/Abstract]) OR (aerobic training[Title/Abstract]) OR (fitness[Title/Abstract]) OR (strength exercise[Title/Abstract]) OR (strength training[Title/Abstract]) OR (resistance exercise[Title/Abstract]) OR (resistance training[Title/Abstract]) OR (sport*[Title/Abstract]) OR (exercis*[Title/Abstract]) OR (exercise intervention*[Title/Abstract]) OR (Motor activity[Title/Abstract])) AND ((nutrition*[Title/Abstract]) OR (paleolithic diet[Title/Abstract]) OR (low carbohydrate diet[Title/Abstract]) OR (carnivore diet[Title/Abstract]) OR (atkins diet[Title/Abstract]) OR (ketogenic diet[Title/Abstract]) OR (keto* diet[Title/Abstract]) OR (elimination diet[Title/Abstract]) OR (elemental diet[Title/Abstract]) OR (experimental diet[Title/Abstract]) OR (fasting[Title/Abstract]) OR (Mediterranean diet[Title/Abstract]) OR (Cretan Mediterranean diet[Title/Abstract]) OR (vege* diet[Title/Abstract]) OR (vegan diet[Title/Abstract]) OR (lacto vegetarian diet[Title/Abstract]) OR (diet*[Title/Abstract])) AND ((psoriasis[Title/Abstract]) OR (joint damage[Title/Abstract]) OR (Spondyloarthropathies[Title/Abstract]) OR (Axial Spondyloarthropathy[Title/Abstract]) OR (Rheumatism[Title/Abstract]) OR (Ankylosing spondylitis[Title/Abstract]) OR (rheumatic disease[Title/Abstract]) OR (axial spondyloarthritis[Title/Abstract]) OR (psoriatic arthritis[Title/Abstract]) OR (rheumatoid arthritis[Title/Abstract]) OR (inflammatory arthritis[Title/Abstract]))) NOT ((animals[MeSH Terms]) NOT (humans[MeSH Terms])))

EMBASE

1 "physical activity intervention*".ab,ti. (4367)
2 "physical activit*".ab,ti. (185317)
3 aerobic training.ab,ti. (4235)
4 fitness.ab,ti. (98330)
5 strength exercise.ab,ti. (800)
6 strength training.ab,ti. (7308)
7 resistance exercise.ab,ti. (7699)
8 resistance training.ab,ti. (11522)
9 "sport*".ab,ti. (118109)
10 "exercis*".ab,ti. (438694)
11 "exercise intervention*".ab,ti. (10936)
12 Motor activity.ab,ti. (17775)
13 1 or 2 or 3 or 4 or 5 or 6 or 7 or 8 or 9 or 10 or 11 or 12 (757311)
14 "nutrition*".ab,ti. (410068)
15 paleolithic diet.ab,ti. (131)
16 low carbohydrate diet.ab,ti. (1787)
17 carnivore diet.ab,ti. (15)
18 atkins diet.ab,ti. (475)
19 ketogenic diet.ab,ti. (5177)
20 "keto* diet".ab,ti. (5228)
21 elimination diet.ab,ti. (1732)
22 elemental diet.ab,ti. (1021)
23 experimental diet.ab,ti. (1213)
24 fasting.ab,ti. (179878)
25 Mediterranean diet.ab,ti. (8638)

26 Cretan Mediterranean diet.ab,ti. (19)
27 "vege* diet".ab,ti. (1972)
28 vegan diet.ab,ti. (687)
29 lacto vegetarian diet.ab,ti. (47)
30 "diet*".ab,ti. (781383)
31 14 or 15 or 16 or 17 or 18 or 19 or 20 or 21 or 22 or 23 or 24 or 25 or 26 or 27 or 28 or 29 or 30 (1216835)
32 psoriasis.ab,ti. (67976)
33 joint damage.ab,ti. (6745)
34 Spondyloarthropathies.ab,ti. (2264)
35 Axial Spondyloarthropathy.ab,ti. (222)
36 Rheumatism.ab,ti. (10263)
37 Ankylosing spondylitis.ab,ti. (24822)
38 rheumatic disease.ab,ti. (9576)
39 axial spondyloarthritis.ab,ti. (5117)
40 psoriatic arthritis.ab,ti. (22200)
41 rheumatoid arthritis.ab,ti. (166664)
42 inflammatory arthritis.ab,ti. (11275)
43 32 or 33 or 34 or 35 or 36 or 37 or 38 or 39 or 40 or 41 or 42 (277838)
44 13 and 31 and 43 (580)
45 limit 44 to human (551)

SportDiscus

((((physical activity intervention*[Title/Abstract]) OR (physical activit*[Title/Abstract]) OR (aerobic training[Title/Abstract]) OR (fitness[Title/Abstract]) OR (strength exercise[Title/Abstract]) OR (strength training[Title/Abstract]) OR (resistance exercise[Title/Abstract]) OR (resistance training[Title/Abstract]) OR (sport*[Title/Abstract]) OR (exercis*[Title/Abstract]) OR (exercise intervention*[Title/Abstract]) OR (Motor activity[Title/Abstract])) AND ((nutrition*[Title/Abstract]) OR (paleolithic diet[Title/Abstract]) OR (low carbohydrate diet[Title/Abstract]) OR (carnivore diet[Title/Abstract]) OR (atkins diet[Title/Abstract]) OR (ketogenic diet[Title/Abstract]) OR (keto* diet[Title/Abstract]) OR (elimination diet[Title/Abstract]) OR (elemental diet[Title/Abstract]) OR (experimental diet[Title/Abstract]) OR (fasting[Title/Abstract]) OR (Mediterranean diet[Title/Abstract]) OR (Cretan Mediterranean diet[Title/Abstract]) OR (vege* diet[Title/Abstract]) OR (vegan diet[Title/Abstract]) OR (lacto vegetarian diet[Title/Abstract]) OR (diet*[Title/Abstract])) AND ((psoriasis[Title/Abstract]) OR (joint damage[Title/Abstract]) OR (Spondyloarthropathies[Title/Abstract]) OR (Axial Spondyloarthropathy[Title/Abstract]) OR (Rheumatism[Title/Abstract]) OR (Ankylosing spondylitis[Title/Abstract]) OR (rheumatic disease[Title/Abstract]) OR (axial spondyloarthritis[Title/Abstract]) OR (psoriatic arthritis[Title/Abstract]) OR (rheumatoid arthritis[Title/Abstract]) OR (inflammatory arthritis[Title/Abstract])))

Table S1: Characteristics of the eligible studies (extracted data)

ID	Design	Participants anthropometric characteristics	Participants disease characteristics	Physical activity/exercise intervention/measurements	Nutrition/diet intervention/measurements	Main outcome
Barone 2018	Epidemiological	RA patients (n=76, age=56.5±8.8 years, BMI=24.9±3.2) PsA patients (n=70, age=55.3±9.1 years, BMI=25.6±3.0) AS patients (n=22, age=51.6±8.8 years, BMI=24.9±2.5)	RA disease duration=10.8±8.2, disease remission 43.4% (CDAI), 44.7% (SDAI), 47.3% (DAS28); PsA disease duration=11.1±8.1, disease remission 36.7% (DASPA); AS disease duration=14.5±8.4, disease remission 59.1% (BASDAI), 50% (ASDAS-CRP), 28.5% (ASDAS-ESR)	Measurements of physical activity level 1.3±0.1 (ratio of total to basal body daily energy expenditure	Measurements of daily calorie and protein intake	Physical activity levels, calorie and protein intake were not associated with sarcopenia in RA PsA, and AS patients
Bilberg 2022	CT	PsA patients (women n=26, men n=15; Age=54 years, IQR 48.5-62; BMI=35.2, IQR 34.1-38.1) Control group obese (women n=31, men n=11; Age=54.5 years, IQR 46.2-60; BMI=38.5, IQR 36.9-41.7)	PsA disease duration=17 years (11-27); DASPA score=15.3 (6.6-29); DAS28-CRP score=2.9 (2.1-3.7); peripheral arthritis 85.4% of patients; axial disease 4.9% of patients; peripheral arthritis and axial disease combination 9.7% of patients	Recommendation for physical activity, ≥ 150 min of moderately intense weekly activity, and reduced amount of sedentary time, for 12 months	Weight loss treatment with very low energy diet (< 800 kcal/day), for 12 months. Four portions of powder dissolved in cold or hot water consumed as shakes or soups, providing a total daily intake of 640 kcal	PsA patients improved SF-36 score from baseline to 6-month ($p<0.01$) and 12-month ($p<0.01$). Baseline score (median)= 35.8 IQR=24.9-46.3; 6-month score (median)= 45.7 IQR=37-51.5; 12-month score (median)= 46.1 IQR=34.5-49.8.

PsA patients had no improvement in hand pain.

PsA patients improved timed to stand test from baseline to 6-month (p<0.01) and 12-month (p<0.01). Baseline score (median)= 26.9 IQR=22.1-35.4; 6-month score (median)= 23.3 IQR=18.5-29.8; 12-month score (median)= 23.2 IQR=19.4-30.4.

PsA patients improved total fat mass (kg) from baseline to 12-month (p<0.01). Baseline (median)= 48.5 IQR=41.7-56.7; 12-month (median)=33.9 IQR=25.9-40.5.

PsA patients reduced total lean mass (kg) from baseline to 12-month (p<0.01). Baseline (median)= 51.9 IQR=45.9-61.8; 12-month (median)=48.3 IQR=43.6-58.3.

						PsA patients improved BMI from baseline to 12-month (p<0.01). Baseline (median)= 35.2 IQR=34.1-38.1; 12-month (median)=30.5 IQR=28-32.9.<br><br>Control group improved BMI from baseline to 12-month (p<0.01). Baseline (median)= 38.5 IQR=36.9-41.7; 12-month (median)=32.6 IQR=30.3-34.8. There was a significant difference in BMI between PA group and controls at the end of the intervention (p=0.02).
Elkan 2011	Epidemiological	RA patient (n=61, women; age=60.8, 95% CI 57.3-64.4 years; BMI=24.2, 95% CI 21.6-26.6; Fat mass %=37.8, 95% CI 35.7-39.9)	RA disease duration (median)=6, IQR 2-15; DAS28 score= 3.3, 95% CI 3-3.6; ESR (mm/h) median= 16, IQR 9-29.	Self-administered of the international physical activity questionnaire (IPAQ) was used to assess average total physical activity during the previous year	Self-administered food-frequency items in the questionnaire (FFQ) asked the patients to report usual frequency of consumption of 88 food items and beverages over the previous year	RA patients who consumed saturated fatty acids and had low level of total physical activity displayed significantly lower levels of HDL, Apolipoprotein A1, the atheroprotective anti-phosphorylcholine antibodies, and significantly higher levels of insulin,

						compared to those who consumed less saturated fatty acids and had high level of total physical activity
Engelhart 1996	CT	RA patients (n=20, 3 men, 17 women). Age median=55, range 34-71 years.	RA disease duration (median)=8, range 1-34; Functional class of RA (median)=I, range I-II; ESR (median, mm)[3]=32, range 2-104.	A 12-week intervention: Dynamic strength and conditioning exercises 3 times per week, 10 minutes warm-up, 20 minutes exercises per day, 5 minutes stretching. On non-exercise days 30 minutes walking	A 12-week intervention: Reduction of 30% of energy intake; 62 gr of high-quality protein intake per day; vitamins and mineral supplements	High-protein-low energy diet in combination with resistance exercise, reduces body weight and preserve body composition and muscle function. Body weight baseline=82.5±2.9 (SEM); Body weight reduction=4.47±0.51 (SEM, p<0.01). Body fat mass baseline=32.4±1.6 (SEM); Body fat mass reduction=2.74±0.84 (SEM, p<0.01). Body fat free mass baseline=50.1±2.1 (SEM); Body fat free mass reduction=1.73±0.61 (SEM, p<0.01)
Garcia-Morales 2020	RCT	Group 1: RA women patients MD+DEP (n=36; Age=51.4±12.4 years, BMI=27.2±3.2).	All patients received conventional disease modifying antirheumatic drugs.	24-week of combined exercise program (aerobic+resistance), 2 times per week, 80-90 minutes per	24-week of MD individualized prescribed according to basal energy expenditure	Analyzed sample size: Group 1 MD+DEP=32 Group 2 DEP=36 Group 3 MD=35 Group 4 control=27

	Group 2: RA women patients DEP (n=37; Age=49.7±11.4 years, BMI=26.5±3.2).<br>Group 3: RA women patients MD (n=40; Age=46.3±13.1 years, BMI=27.2±3.6).<br>Group 4: RA women patients control (n=31; Age=49.1±12.1 years, BMI=27.1±4.2).	Group 1: RA women patients MD+DEP (disease duration median=17, IQR 7-21 years; Pain VAS median =6, IQR 4.3-7, DAS28=2.47±0.79; CRP mg/dL median=0.65, IQR 0.25-1.40; ESR mm/h median=13, IQR 7-26).<br>Group 2: RA women patients DEP (disease duration median=14, IQR 5-20.5 years; Pain VAS median =6, IQR 4-8, DAS28=2.67±0.90; CRP mg/dL median=0.62, IQR 0.25-1.19; ESR mm/h median=10, IQR 6.5-16).<br>Group 3: RA women patients MD (disease duration median=12, IQR 6-19.5 years; Pain VAS median =5, IQR 2-8, DAS28=2.2±1.06; CRP mg/dL median=0.28, IQR 0.11-0.55; ESR mm/h median=9, IQR 3-19).<br>Group 4: RA women patients control (disease duration median=8, IQR 4-24 years; Pain VAS median =5, IQR 3.8-7.3,	session, 20 minutes of static bicycle 65-85% of maximum heart rate, 20 minutes of resistance exercise and 20 minutes of recreational games (basketball, soccer, volleyball etc.). General intensity of exercise according to ASCM.	estimated using Harris and Benedict's equation. 50% carbohydrates, 30% fats, and 20%proteins.	MD+DEP group compared to control group improved<br>1. Physical function MD+DEP=77.5, 95% CI 51.3-88.8; Control=70, 95% CI 45-90, p<0.01.<br>2. Global health MD+DEP=50, 95% CI 40-70; Control=45, 95% CI 29.8-67.5, p<0.01.<br><br>MD+DEP group improved from baseline<br>1. Physical function MD+DEP baseline=50, 95% CI 31.2-68.7; MD+DEP 24-week=77.5, 95% CI 51.3-88.8, p<0.01.<br>2. Vitality MD+DEP baseline=50, 95% CI 31.2-60; MD+DEP 24-week=50, 95% CI 40-75, p<0.01.<br>3. Mental health MD+DEP baseline=66, 95% CI 48-83; MD+DEP 24-week=74, 95% CI 60-92, p<0.01.<br>4. Social function MD+DEP baseline=61.2, 95% CI 35-79.3;

			DAS28=2.59±0.88; CRP mg/dL median=0.34, IQR 0.19-0.53; ESR mm/h median=13, IQR 9.5-21.5).			MD+DEP 24-week=77.5, 95% CI 56.9-100, p<0.01.<br>5. Global punctuation<br>MD+DEP baseline=53.4, 95% CI 34.5-64.2;<br>MD+DEP 24-week=67.8, 95% CI 46.4-82.4, p<0.01.<br>6. HAQ-DI<br>MD+DEP baseline=1.25, 95% CI 0.75-1.5;<br>MD+DEP 24-week=0.81, 95% CI 0.41-1.22, p<0.01.<br><br>MD+DEP group worsen from baseline bodily pain: MD+DEP baseline=41, 95% CI 22-61.7; MD+DEP 24-week=61.5, 95% CI 44.3-83, p<0.01.
Garner 2018	RCT	RA patients<br>1. Standard care group (n=14, 10 women), Age=49±14 years; BMI=27.2±7.3; Waist-to-hip ratio=0.87±0.07.<br><br>2. Intervention group (n=14, 13 women), Age=45±10 years; BMI=25.4±4.3; Waist-to-hip ratio=0.83±0.06.	1. Standard care group Swollen joint count (28 joints)=12.4±8.4; Tender joint count (28 joints)=14.1±8; Patient Global Evaluation Score (0–100 VAS)=45.7±27.1; ESR (mm/h)=21.6±18.1; CRP (mg/L)=18.6±30.4; DAS28=5.5±1.4; HAQ score=1±0.9.<br><br>2. Intervention group	6-month Nutrition counselling: Patients completed the National Cancer Institute's food frequency questionnaire, adapted to the Canadian food supply (Csizmadi et al., 2007). They then reviewed the Dietary Reference Intake	6-month Physical activity counselling: Review of patients' current physical activity levels and the results of their fitness tests, and then received instruction on national physical activity guidelines for their age group (Canadian Society for Exercise	Analyzed sample size: Standard care group=10 Intervention group=13 Both the intervention and the standard care groups showed an improvement in Swollen joint count (28 joints), Tender joint count (28 joints), Global Evaluation Score (0–100 VAS), ESR, DAS28 score, HAQ score, BMI,

			Swollen joint count (28 joints)=3.6±4.5; Tender joint count (28 joints)=4.4±4.9; Patient Global Evaluation Score (0–100 VAS)=35.7±17; ESR (mm/h)=12.8±9.6; CRP (mg/L)=3.1±5.4; DAS28=3.5±1.1; HAQ score=0.6±0.4.	recommendations for their age group (Institute of Medicine, 2010) and had specific questions answered pertaining to their particular diet concerns.	Physiology, 2016), along with specific exercises to improve their fitness level.	waist-to-hip-ratio, LDL, blood pressure. No statistical differences found between groups, at the end of the intervention
Gordon 2002	Single arm interventional studies	RA patients (n=22, 20 women; Age median=50, range 32-80 years)	Disease duration median=4, range 0.5-13 years; Ritchie articular index median=6, range 0-24; Duration of morning stiffness median=60, range 0-960 minutes; ESR (mm/h) median=24, range 1-72; CRP (mg/l) median=20, range 6-85; HAQ score median=1.75, range 0.5-2.875.	48-week of dietary advice was given verbally on low fat, high fiber diets containing at least five portions of fruit and vegetables a day. If a specific dietary problem was identified, review was arranged with a dietician	48-week of counselling of taking regular exercise, 20 minutes 3 times a week in a swimming pool	RA patients improved ESR, CRP and Ritchie articular index at the end of the intervention compared to baseline: 1. ESR (mm/h) baseline median=24, range 1-72; ESR (mm/h) 48-week median=22, range 2-75, $p>0.05$. 2. CRP (mg/l) baseline median=20, range 6-85; CRP (mg/l) 48-week median=8, range 6-41, $p<0.01$. 3. Ritchie articular index baseline median=6, range 0-24; Ritchie articular index 48-week median=4, range 0-16, $p>0.05$.

						No changes in HAQ score and Haemoglobin were found.
Matsunaga 2021	Epidemi ological	Participants from the general population (n=11768)	Data not shown	Data from National Health and Nutrition Examination Survey (NHANES)	Data from National Health and Nutrition Examination Survey (NHANES)	Healthy eating index total scores were not associated with self-reported RA, adjusted for physical activity
Mikkelsen 2015	CT	RA patients (n=13); Age=56±14 years; BMI=25±4; Waist-to-hip ratio=0.92±0.06; Fat mass %=31.3±3 (SEM); Lean body mass (kg)=44.5±1.3 (SEM)<br>Controls (n=13); Age=57±15 years; BMI=25±5; Waist-to-hip ratio=0.88±0.08; Fat mass %=31±2.9 (SEM); Lean body mass (kg)=45.2±2.1 (SEM)	RA patients (n=6) DAS28 score=2.6±1	A protein drink consisting of 0.5 g intact whey protein isolate (Lacprodan-9224, Arla Foods Ingredients, Viby, Denmark)/kg lean body mass (12.5 % enriched with ring-13C6- phenylalanine) dissolved in 190 ml water.<br>Total amount consumed by the RA patients 25.3±0.7 (SEM) gr, and by the controls 25.7±1.2 (SEM) gr	Acute exercise: Unilateral leg extension, 8x10 repetitions at 70% of 1 repetition maximum.	Muscle protein synthesis and transcriptional regulation can be stimulated with both protein intake and physical exercise in patients with RA to a similar degree as in healthy individuals. The myofibrillar protein synthesis was enhanced in response to protein intake ($p<0.05$) and was further increased when combined with heavy resistance exercise ($p<0.001$).
Pineda-Juarez 2022	RCT	Group 1: RA women patients MD+DEP (n=34; Age=49.5±13.6 years).<br>Group 2: RA women patients DEP (n=34; Age=47.1±11 years).	Group 1: RA women patients MD+DEP (disease duration median=16.5, IQR 9.5-21 years; DAS28=2.4±0.88; HAQ-DI median=1.1, IQR 0.6-1.5).	24-week of combined exercise program (aerobic+resistance), 2 times per week, 18 sessions; 20 minutes of static bicycle 65-85% of maximum	24-week of MD individualized prescribed according to basal energy expenditure estimated using Harris and Benedict's	Analyzed sample size:<br>Group 1 MD+DEP=34<br>Group 2 DEP=34<br>Group 3 MD=38

		Group 3: RA women patients MD (n=38; Age=48.2±13.2 years).	Group 2: RA women patients DEP (disease duration median=12, IQR 6-20 years; DAS28=2.5±0.88; HAQ-DI median=0.7, IQR 0.3-1.2). Group 3: RA women patients MD (disease duration median=12.5, IQR 7.5-19 years; DAS28=2.4±1.2; HAQ-DI median=0.5, IQR 0-0.9).	heart rate; 7-10 different resistance exercises, 8-15 repetitions; recreational games (basketball, soccer, volleyball etc.). General intensity of exercise according to ASCM.	equation. 50% carbohydrates, 30% fats, and 20%proteins.	Combination of DEP+MD reduced HAQ-DI ($p<0.01$). HAQ-DI baseline median=1.2, IQR 0.6-1.5 HAQ-DI 24-week median=0.8, IQR 0.4-1.1. No changes in body weight and waist circumference
Stavropoulos-Kalinoglou 2009	Epidemiological	RA patients (n=150, women n=102); Men: Age median=60, IQR 59-64 years; BMI median=27.5, IQR 24.4-29.6; Body fat median=26.1, IQR 23.7-30.4%. Women: Age median=59, IQR 55-64 years; BMI median=25.5, IQR 23.8-27; Body fat median=34.8, IQR 30-40.4%.	Men: Disease duration median=7, IQR 4-12; DAS28 median=4.4, IQR 2.6-5.3; HAQ median=1.4, IQR 0.6-1.8; RF positive= 68.6%; DMARD= 88.2%. Women: Disease duration median=9, IQR 5-14; DAS28 median=3.8, IQR 2.9-5.1; HAQ median=1.6, IQR 1-1.9; RF positive= 70.2%; DMARD= 90.1%.	IPAQ long form measurements	3-day dietary recall questionnaire	Physical activity and energy intake were not associated with HAQ, DAS28, body fat, BMI, Interleukin-1β, Interleukin-6, Tumor necrosis factor alpha, ESR and CRP.

Key: Values are presented as means and standard deviations, unless is otherwise specified. RA= Rheumatoid arthritis; PsA= Psoriatic arthritis; AS= Ankylosing spondylitis; BMI= Body mass index; CDAI= clinical disease activity index; SDAI= simplified disease activity index; DAS28= disease activity score 28; DASPA= Disease Activity Index for Psoriatic Arthritis; BASDAI= Bath Ankylosing Spondylitis Disease Activity Index; ASDAS-CRP= Ankylosing Spondylitis Disease Activity Score-CRP; ASDAS-ESR= Ankylosing Spondylitis Disease Activity Score-ESR; CT= controlled trial; IQR=Interquartile range; SF-36= Short form (36) health survey; BMI=Body mass index; CI=confidence interval;

OR=Odds ratio; NHANES= Nutrition and Health Examination Surveys; ESR= erythrocyte sedimentation rate; HDL=High density lipoprotein; SEM=Standard error of the mean; RCT=Randomized controlled trial; MD=Mediterranean diet; DEP=Dynamic exercise program; VAS=Visual analog scale; ASCM=American college of sports medicine; HAQ-DI= Health Assessment Questionnaire Disability Index; SEM=Standard error of the mean; RF= Rheumatoid factor; DMARD= disease-modifying anti-rheumatic drug; IPAQ=International physical activity questionnaire; CRP=C reactive protein.

Figure S1: PRISMA flow diagram

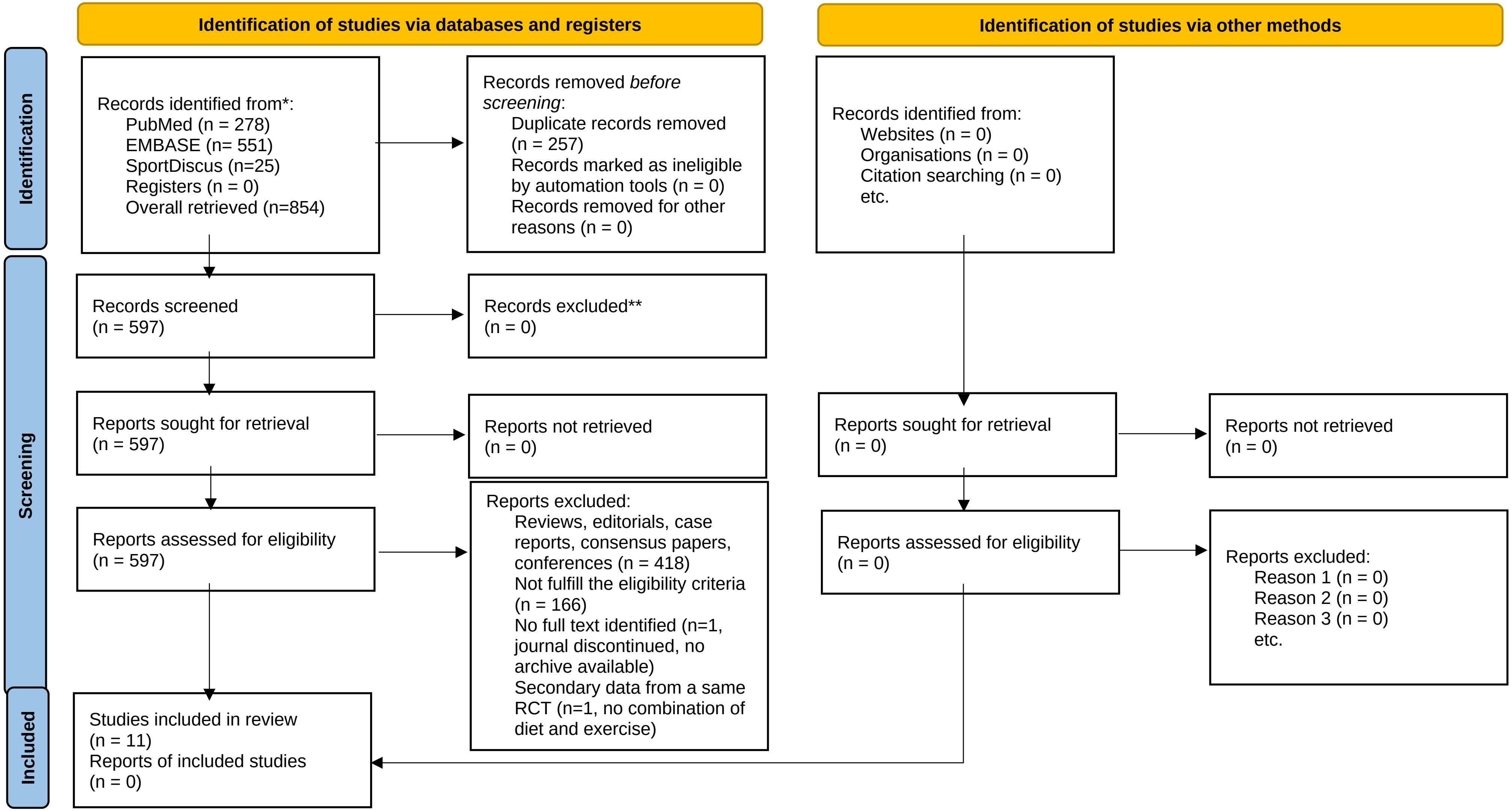

*Consider, if feasible to do so, reporting the number of records identified from each database or register searched (rather than the total number across all databases/registers).
**If automation tools were used, indicate how many records were excluded by a human and how many were excluded by automation tools

Figure S2: Forest plot of the effect of combined intervention of diet/nutrition and physical activity/exercise on weight (kg), in RA patients

	Exercise-diet/nutrition			Control				Mean Difference
**Study or Subgroup**	**Mean**	**SD**	**Total**	**Mean**	**SD**	**Total**	**Weight**	**IV, Random, 95% CI**
Engelhart 1996	78.3	4.35	20	82.5	12.97	20	34.3%	−4.20 [−10.20, 1.80]
Pineda–Juarez 2022	63.63	6.15	34	64.83	11.33	34	65.7%	−1.20 [−5.53, 3.13]
**Total (95% CI)**			**54**			**54**	**100.0%**	**−2.23 [−5.74, 1.28]**

Heterogeneity: $Tau^2 = 0.00$; $Chi^2 = 0.63$, df = 1 (P = 0.43); $I^2 = 0\%$
Test for overall effect: Z = 1.24 (P = 0.21)

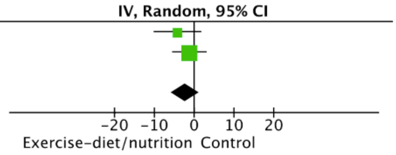

Figure S3: Forest plot of the effect of combined intervention of diet/nutrition and physical activity/exercise on CRP (mg/L), in RA patients

	Exercise-diet/nutrition			Control				Mean Difference
**Study or Subgroup**	**Mean**	**SD**	**Total**	**Mean**	**SD**	**Total**	**Weight**	**IV, Random, 95% CI**
Gordon 2002	18.33	25.93	22	37	58.52	22	19.4%	−18.67 [−45.42, 8.08]
Garner 2017	1.56	0.09	13	2.8	0.03	10	80.6%	−1.24 [−1.29, −1.19]
**Total (95% CI)**			**35**			**32**	**100.0%**	**−4.61 [−18.11, 8.88]**

Heterogeneity: $Tau^2 = 58.79$; $Chi^2 = 1.63$, df = 1 (P = 0.20); $I^2 = 39\%$
Test for overall effect: Z = 0.67 (P = 0.50)

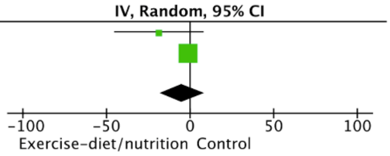

Table S2: Detailed GRADE analysis for the meta-analyses performed

S3.1	Evaluation components to lower quality					
**Outcome**	**Methodological design**	**Risk of bias**	**Inconsistency of results**	**Indirectness**	**Imprecision**	**Publication bias**
Diet/nutrition and physical activity/exercise HAQ score vs. Control	Randomized controlled trials and controlled trials: Moderate quality	15 (63%) of the 24 components of the included studies display low risk of bias, eight (33%) components display some concerns, and one (4%) high risk of bias. No downgrade	Even though we used a random effect model meta-analysis, we consider heterogeneity as an index of inconsistency. I2=90%, p<0.01, substantial heterogeneity. Downgrade 1 level	All studies do display as a primary aim, very similar to the systematic review aim. Therefore, the available evidence is applicable to our research question. No downgrade	1. The overall sample size is low (n=199), therefore, the optimal information size is not met. 2. The confidence interval of the overall effect excludes the "favor control" values. The confidence interval represents the true underlying effect. No downgrade	The studies in this meta-analysis do not suffer from important limitations, the evidence is direct and consistent. No major funding from the industry. No publication bias in the funnel plots. No downgrade
Diet/nutrition and physical activity/exercise ESR vs. Control	Randomized controlled trials and controlled trials: Moderate quality	The included studies display 50% of low risk of bias, 42% some concerns and 8% high risk of bias. No downgrade	Even though we used a random effect model meta-analysis, we consider heterogeneity as an index of inconsistency. I2=0%, p>0.05, no heterogeneity. No downgrade	All studies do display as a primary aim, very similar to the systematic review aim. Therefore, the available evidence is applicable to our research question. No downgrade	1. The overall sample size is low (n=67), therefore, the optimal information size is not met. 2. The confidence interval of the overall effect does not exclude the "favor control" values. The confidence interval represents the true underlying effect. Downgrade 1 level	The studies in this meta-analysis do not suffer from important limitations, the evidence is direct and consistent. No major funding from the industry. No publication bias in the funnel plots. No downgrade

S3.2	Evaluation components to higher quality		
**Outcome**	**Large effect**	**Dose response**	**Confounding**
Diet/nutrition and exercise/physical activity HAQ score vs. Control	Given that the data are skewed, we converted SMD to Odds ratio (OR) using the equation LogOR= (π/√3)*SMD and we converted the LogOR into Risk Ratio (RR) using the equation RR= OR/ (1-Absolute Control Risk)*(1-OR). We	No robust evidence for a dose response effect. No upgrade	We found no confounding factors that indicate upgrading

	assumed an absolute control risk reduction of 20% (ACR=0.2). The outcome showed a RR<2. No upgrade		
Diet/nutrition and exercise/physical activity ESR vs. Control	Given that the data are skewed, we converted SMD to Odds ratio (OR) using the equation LogOR= $(\pi/\sqrt{3})$*SMD and we converted the LogOR into Risk Ratio (RR) using the equation RR= OR/ (1-Absolute Control Risk)*(1-OR). We assumed an absolute control risk reduction of 20% (ACR=0.2). The outcome showed a RR<2. No upgrade	No robust evidence for a dose response effect. No upgrade	We found no confounding factors that indicate upgrading

Table S3: PRISMA checklist

Section and Topic	Item #	Checklist item	Location where item is reported
**TITLE**			
Title	1	Identify the report as a systematic review.	Page 1
**ABSTRACT**			
Abstract	2	See the PRISMA 2020 for Abstracts checklist.	Page 1
**INTRODUCTION**			
Rationale	3	Describe the rationale for the review in the context of existing knowledge.	Pages 1-2
Objectives	4	Provide an explicit statement of the objective(s) or question(s) the review addresses.	Pages 1-2
**METHODS**			
Eligibility criteria	5	Specify the inclusion and exclusion criteria for the review and how studies were grouped for the syntheses.	Pages 2-3
Information sources	6	Specify all databases, registers, websites, organisations, reference lists and other sources searched or consulted to identify studies. Specify the date when each source was last searched or consulted.	Page 2
Search strategy	7	Present the full search strategies for all databases, registers and websites, including any filters and limits used.	Page 2, supplement
Selection process	8	Specify the methods used to decide whether a study met the inclusion criteria of the review, including how many reviewers screened each record and each report retrieved, whether they worked independently, and if applicable, details of automation tools used in the process.	Page 2
Data collection process	9	Specify the methods used to collect data from reports, including how many reviewers collected data from each report, whether they worked independently, any processes for obtaining or confirming data from study investigators, and if applicable, details of automation tools used in the process.	Page 3
Data items	10a	List and define all outcomes for which data were sought. Specify whether all results that were compatible with each outcome domain in each study were sought (e.g. for all measures, time points, analyses), and if not, the methods used to decide which results to collect.	Pages 3-4
	10b	List and define all other variables for which data were sought (e.g. participant and intervention characteristics, funding sources). Describe any assumptions made about any missing or unclear information.	Pages 3-4
Study risk of bias assessment	11	Specify the methods used to assess risk of bias in the included studies, including details of the tool(s) used, how many reviewers assessed each study and whether they worked independently, and if applicable, details of automation tools used in the process.	Page 3

Section and Topic	Item #	Checklist item	Location where item is reported
Effect measures	12	Specify for each outcome the effect measure(s) (e.g. risk ratio, mean difference) used in the synthesis or presentation of results.	Pages 3-4
Synthesis methods	13a	Describe the processes used to decide which studies were eligible for each synthesis (e.g. tabulating the study intervention characteristics and comparing against the planned groups for each synthesis (item #5)).	Pages 3-4
	13b	Describe any methods required to prepare the data for presentation or synthesis, such as handling of missing summary statistics, or data conversions.	Pages 3-4
	13c	Describe any methods used to tabulate or visually display results of individual studies and syntheses.	Pages 3-4 & 6-7
	13d	Describe any methods used to synthesize results and provide a rationale for the choice(s). If meta-analysis was performed, describe the model(s), method(s) to identify the presence and extent of statistical heterogeneity, and software package(s) used.	Pages 3-4 & 6-7
	13e	Describe any methods used to explore possible causes of heterogeneity among study results (e.g. subgroup analysis, meta-regression).	Pages 3-4
	13f	Describe any sensitivity analyses conducted to assess robustness of the synthesized results.	Not applicable
Reporting bias assessment	14	Describe any methods used to assess risk of bias due to missing results in a synthesis (arising from reporting biases).	Page 3
Certainty assessment	15	Describe any methods used to assess certainty (or confidence) in the body of evidence for an outcome.	Page 4
**RESULTS**			
Study selection	16a	Describe the results of the search and selection process, from the number of records identified in the search to the number of studies included in the review, ideally using a flow diagram.	Page 4
	16b	Cite studies that might appear to meet the inclusion criteria, but which were excluded, and explain why they were excluded.	Page 4
Study characteristics	17	Cite each included study and present its characteristics.	Page 4
Risk of bias in studies	18	Present assessments of risk of bias for each included study.	Pages 4-6
Results of individual studies	19	For all outcomes, present, for each study: (a) summary statistics for each group (where appropriate) and (b) an effect estimate and its precision (e.g. confidence/credible interval), ideally using structured tables or plots.	Pages 6-8
Results of	20a	For each synthesis, briefly summarise the characteristics and risk of bias among contributing studies.	Pages 6-8

Section and Topic	Item #	Checklist item	Location where item is reported
syntheses	20b	Present results of all statistical syntheses conducted. If meta-analysis was done, present for each the summary estimate and its precision (e.g. confidence/credible interval) and measures of statistical heterogeneity. If comparing groups, describe the direction of the effect.	Page 8
	20c	Present results of all investigations of possible causes of heterogeneity among study results.	Not applicable
	20d	Present results of all sensitivity analyses conducted to assess the robustness of the synthesized results.	Not applicable
Reporting biases	21	Present assessments of risk of bias due to missing results (arising from reporting biases) for each synthesis assessed.	Not applicable
Certainty of evidence	22	Present assessments of certainty (or confidence) in the body of evidence for each outcome assessed.	Page 8
**DISCUSSION**			
Discussion	23a	Provide a general interpretation of the results in the context of other evidence.	Page 9
	23b	Discuss any limitations of the evidence included in the review.	Page 10
	23c	Discuss any limitations of the review processes used.	Page 10
	23d	Discuss implications of the results for practice, policy, and future research.	Page 11
**OTHER INFORMATION**			
Registration and protocol	24a	Provide registration information for the review, including register name and registration number, or state that the review was not registered.	Page 2
	24b	Indicate where the review protocol can be accessed, or state that a protocol was not prepared.	Page 2
	24c	Describe and explain any amendments to information provided at registration or in the protocol.	Page 10
Support	25	Describe sources of financial or non-financial support for the review, and the role of the funders or sponsors in the review.	Page 11
Competing interests	26	Declare any competing interests of review authors.	Page 11
Availability of data, code and other materials	27	Report which of the following are publicly available and where they can be found: template data collection forms; data extracted from included studies; data used for all analyses; analytic code; any other materials used in the review.	Page 11
